# Pre-B-cell colony-enhancing factor protects against apoptotic neuronal death and mitochondrial damage in ischemia

**DOI:** 10.1038/srep32416

**Published:** 2016-08-31

**Authors:** Xiaowan Wang, Hailong Li, Shinghua Ding

**Affiliations:** 1 Dept. of Bioengineering, University of Missouri, Columbia, MO 65211; 2 Dalton Cardiovascular Research Center University of Missouri, Columbia, MO 65211.

## Abstract

We previously demonstrated that Pre-B-cell colony-enhancing factor (PBEF), also known as nicotinamide phosphoribosyltransferase (NAMPT), the rate-limiting enzyme in mammalian NAD^+^ biosynthesis pathway, plays a brain and neuronal protective role in ischemic stroke. In this study, we further investigated the mechanism of its neuroprotective effect after ischemia in the primary cultured mouse cortical neurons. Using apoptotic cell death assay, fluorescent imaging, molecular biology, mitochondrial biogenesis measurements and Western blotting analysis, our results show that the overexpression of PBEF in neurons can significantly promote neuronal survival, reduce the translocation of apoptosis inducing factor (AIF) from mitochondria to nuclei and inhibit the activation of capase-3 after glutamate-induced excitotoxicity. We further found that the overexpression of PBEF can suppress glutamate-induced mitochondrial fragmentation, the loss of mitochondrial DNA (mtDNA) content and the reduction of PGC-1 and NRF-1 expressions. Furthermore, these beneficial effects by PBEF are dependent on its enzymatic activity of NAD^+^ synthesis. In summary, our study demonstrated that PBEF ameliorates ischemia-induced neuronal death through inhibiting caspase-dependent and independent apoptotic signaling pathways and suppressing mitochondrial damage and dysfunction. Our study provides novel insights into the mechanisms underlying the neuroprotective effect of PBEF, and helps to identify potential targets for ischemic stroke therapy.

Stroke is a leading cause of human disability and death and has a large impact on public health. Focal ischemic stroke (FIS) is a major form of stroke and accounts for approximately 80% of all human strokes. FIS causes primary neuronal death in the ischemic core, and leads to delayed neuronal death in the penumbra. Though various mechanisms by which FIS causes neuronal death and brain damage have been suggested from experimental studies, clinically there have limited strategies for stroke therapy. Thus exploring new molecular pathways that can reduce neuronal death and improve stroke outcomes is important to identify potential therapeutic targets for translational research on stroke therapy.

Brain is a highly metabolically active organ and especially sensitive to an ischemic insult. Energy depletion is the main trigger for rapid necrotic neuronal death in the ischemic core and more delayed apoptosis in the penumbra after a FIS^1^. Thus rescue or compensation for energy metabolism is an important strategy to ameliorate neuronal death and brain damage in FIS. Pre-B-cell colony-enhancing factor (PBEF), also known as nicotinamide phosphoribosyltransferase (NAMPT), is the rate-limiting enzyme in the salvage pathway of mammalian nicotinamide adenine dinucleotide (NAD^+^) biosynthesis through converting nicotinamide (NAM) to nicotinamide mononucleotide (NMN)^2^,^3^,^4^. The major cellular functions of NAD^+^ and its derivative compound NADH include modulating cellular energy metabolism and homeostasis, but NAD^+^ is also a substrate of some NAD^+^ -consuming enzymes such as sirtuins (Sirt1-7) and Poly (ADP-Ribose) Polymerases (PARP)^5^. Previously, we demonstrated for the first time that PBEF was highly expressed in neurons, but not in glial cells, in the mouse brain under normal conditions, and global PBEF heterozygous knockout mice had much larger infarction than wild type (WT) mice when subjected to photothrombosis (PT)-induced FIS^6^. Using *in vitro* ischemia models of primary cultured neurons, we further demonstrated that the overexpression of WT PBEF, but not mutant PBEF lacking enzymatic activity, can reduce neuronal death and inhibit mitochondrial membrane potential (MMP) depolarization after glutamate excitotoxicity^7^. Consistently, the overexpression of PBEF by lentivirus reduces neuronal death and brain damage through Sirt1-dependent AMPK pathway in *in vitro* and *in vivo* ischemia models^8^. Induction of autophagy at the early stage of ischemia also contributes to PBEF-mediated neuroprotection^9^. A recent study reported that overexpression of PBEF in neurons in transgenic mice ameliorates white matter injury after middle cerebral artery occlusion (MCAo) through promoting extracellular PBEF levels^10^, on the other hand, global PBEF overexpression in transgenic mice improves regenerative neurogenesis to promote brain recovery after MCAo^11^. Thus, these studies indicate that PBEF can exert a neuronal and brain protective effect in ischemia through different mechanisms.

It is known that ischemia causes the reduction of NAD^+^ levels^6^,^7^,^12^, and supplementation and maintenance of NAD^+^ can protect against neuronal death in ischemia *in vitro* and *in vivo*^7^,^13^,^14^,^15^. NAD^+^ is also highly correlated with mitochondrial biogenesis^15^,^16^,^17^,^18^. Our results that PBEF can inhibit MMP depolarization and NAD^+^ can suppress mitochondrial fragmentation and the impairment of mitochondrial biogenesis after glutamate excitotoxicity suggest that PBEF may play a critical role in suppressing ischemia-induced mitochondrial damage and biogenesis impairment^7^,^15^. However, the mechanism by which PBEF exerts a neuronal protective effect during ischemic injury in the context of mitochondrial dysfunction has not been studied. In the current study, using *in vitro* ischemia models of glutamate excitotoxicity and oxygen glucose deprivation (OGD) of primary cultured neurons to mimic penumbral conditions in *in vivo* focal ischemia models, we determined the effect of PBEF on apoptotic cell death, apoptotic inducing factor (AIF) translocation, caspase-3 activation, mitochondrial fragmentation and biogenesis. Our study provides evidence that PBEF can protect neurons in ischemia through suppressing caspase-independent and dependent signaling pathways and mitochondrial damage and dysfunction.

## Results

### PBEF reduces apoptotic neuronal death after glutamate excitotoxicity

**Figure 1:**
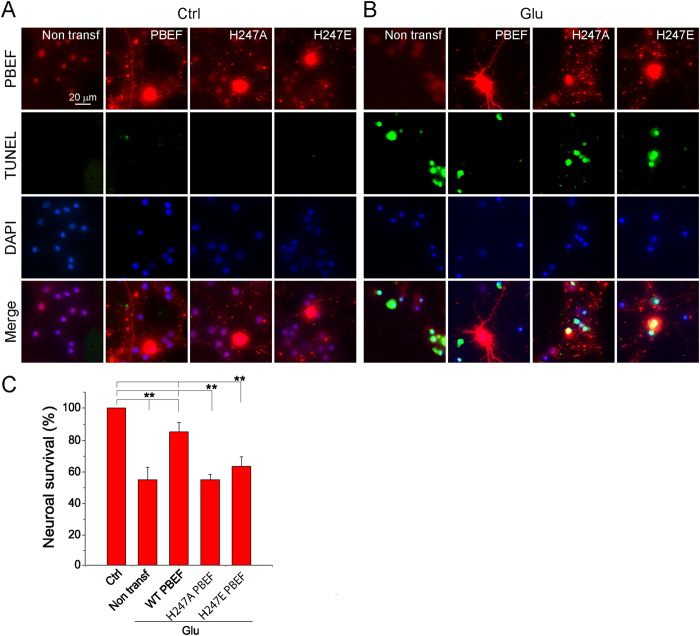
Overexpression of PBEF ameliorates neuronal apoptosis after glutamate excitotoxicity. (**A,B**) Fluorescent images showing the triple staining of TUNEL, PBEF and DAPI in control neurons (**A**) and neurons treated with 30 μM glutamate together with 3 μM glycine for 24 h (**B**). Neuronal cultures were transfected by WT, H247A and H247E PBEF as indicated before the glutamate treatment. During imaging in (**A**), we adjusted exposure time and light power to avoid PBEF signal saturation in transfected neurons, so the endogenous PBEF signals in non-transfected neurons are weak but are visible. (**C**) Neuronal survival rates under different conditions. Notice neurons overexpressed with WT PBEF have much higher survival rate than non-transfected neurons and neurons overexpressed with H247A and H247E PBEF after glutamate stimulation. Data were analyzed from n = 3 independent experiments. **P < 0.01 versus Ctrl and WT PBEF with glutamate stimulation (Glu), ANOVA test.

We initially determined the effect of PBEF on apoptotic neuronal death after ischemia using an *in vitro* ischemic model of glutamate excitotoxicity of primary cultured mouse cortical neurons^7^,^15^. Primary neurons were transfected with DNA plasmids to overexpress WT PBEF or mutant PBEF without NAD^+^ synthetic activity (*i.e.*, H247A and H247E mutants). Two days after transfection, the neurons were then treated with 30 μM glutamate and 3 μM glycine for 24 h, and fixed for double TUNEL and PBEF immunostaining. The transfected neurons could be identified by the enhanced immunofluorescent signal of PBEF. TUNEL+ apoptotic neurons exhibited condensed, shrunken and fragmented nuclei. Our results show that the number of TUNEL+ cells increased significantly after glutamate stimulation (Fig. 1A,B), but much less number of TUNEL+ cells was observed in neurons transfected with WT PBEF than in neurons transfected with H247A and H247E mutants or in neurons without transfection. Quantitative analyses of the survival rates indicate that non-transfected neurons had only a survival rate of 54.4 ± 8.0% after glutamate stimulation, and neurons overexpressing WT PBEF had a survival rate of 84.7 ± 6.0% (Fig. 1C), while neurons overexpressing H247A or H247E PBEF had comparable survival rates with non-transfected neurons. We also tested whether NAM, the substrate of PBEF, had a similar effect on apoptotic neuronal death. Our data show that 15 mM exogenous NAM significantly decreased apoptotic neuronal death (Supl Fig. 1). Thus, our results indicate that PBEF with NAD^+^ synthesis activity can reduce apoptotic neuronal death after glutamate excitotoxicity.

### PBEF overexpression inhibits glutamate-induced AIF translocation

**Figure 2:**
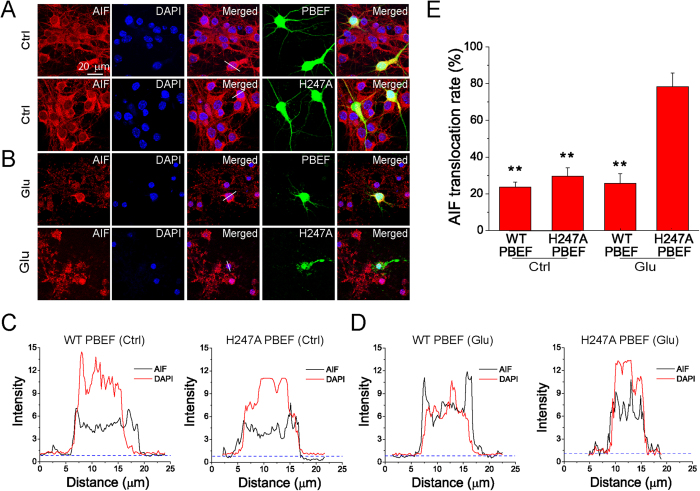
Overexpression of PBEF prevents AIF translocation from mitochondria to the nucleus after glutamate excitotoxicity. (**A,B**) Confocal fluorescent images showing the triple staining of AIF, PBEF and DAPI in neurons under normal (**A**) and glutamate excitotoxicity (**B**) conditions. Neurons co-transfected by WT PBEF or H247A PBEF with EGFP and were treated with 100 μM glutamate together with 10 μM for 3 h. Transfected neurons were identified by EGFP. Translocation of AIF from mitochondria to nuclei is illustrated by the overlap of AIF (red) and DAPI (blue) signals. **(C,D**) Linescan analysis of AIF and DAPI fluorescence from the neurons indicated in (**A,B**). The fluorescence was normalized to the background. Notice the prevention of glutamate-induced AIF translocation by WT PBEF overexpression. (**E**) Summary of AIF translocation after glutamate stimulation. Data was quantified by the number of cells with overlap of AIF and DAPI among the total number of cells determined by DAPI staining. n = 3 three independent experiments. **P < 0.01 versus H247A PBEF with Glu, ANOVA test.

Ischemic stroke leads to NAD^+^ depletion, subsequent translocation of AIF from mitochondria to nucleus^19^, and eventually caspase-independent apoptotic cell death^20^. To test if PBEF could inhibit AIF translocation, primary cultured neurons were co-transfected with WT or H247A PBEF and EGFP (as a marker for transfected cells)^7^. The effects of WT and the mutant PBEF on AIF translocation after glutamate excitotoxicity were evaluated using AIF immunostaining and confocal microscopy. Without glutamate stimulation, AIF was largely localized in the cytoplasm in neurons overexpressing WT PBEF or H247A mutant (Fig. 2A); after 100 μM glutamate stimulation, AIF was still localized in the cytoplasm in neurons overexpressing WT PBEF, but it translocated to the nuclei in neurons overexpressing H247A PBEF (Fig. 2B). Linescan analyses clearly show that under control conditions AIF levels were higher in the cytoplasm than in the nuclei in the neurons overexpressing either WT or mutant PBEF (Fig. 2C), while after glutamate stimulation AIF levels were higher in the nuclei than in the cytoplasm of neurons overexpressing mutant PBEF, but AIF distribution pattern remained the same as control conditions for neurons overexpressing WT PBEF (Fig. 2D). Overall, neurons overexpressing WT PBEF exhibited a dramatic reduction of AIF translocation rate as compared with neurons overexpressing the mutant PBEF after glutamate excitotoxicity, but AIF translocation rates were similar between neurons either overexpressing WT or mutant PBEF under the control conditions (Fig. 2E). Consistently, supplementation of NAM significantly attenuated glutamate-induced AIF translocation (Supl Fig. 2). Together, we found that PBEF can inhibit glutamate-induced AIF translocation through NAD^+^ synthesis.

### PBEF overexpression by viral infection promotes cell viability and reduces caspase-3 activation after glutamate excitotoxicity and OGD

To better investigate the mechanisms of neuroprotective effect by PBEF in ischemia, primary cultured neurons were infected with serotype 2/9 adeno-associated virus (AAV2/9) vectors, *i.e.*, AAV2/9-CMV-PBEF vectors that encode WT PBEF with a His-tag at C-terminal under the control of CMV promoter at 3 DIV. We used this serotype AAV vectors as it has been proved effective for overexpressing transgenes in the primary cultured neurons^21^. PBEF expression was examined 7 days later with immunostaining and Western blot analysis of His-tag and PBEF. Infected neuronal cultures exhibited much higher intensity of PBEF immunofluorescent signals than non-infected neuronal cultures (Supl Fig. 3A). Similarly, His-tag immunoreactivity was detected in infected neurons, but not in non-infected neurons (Supl Fig. 3B). Western blotting analysis revealed a remarkable increase in PBEF levels by viral infection (Supl Fig. 3C). Thus, a significant overexpression of PBEF can be achieved in primary cortical neuronal cultures using AAV infection.

**Figure 3:**
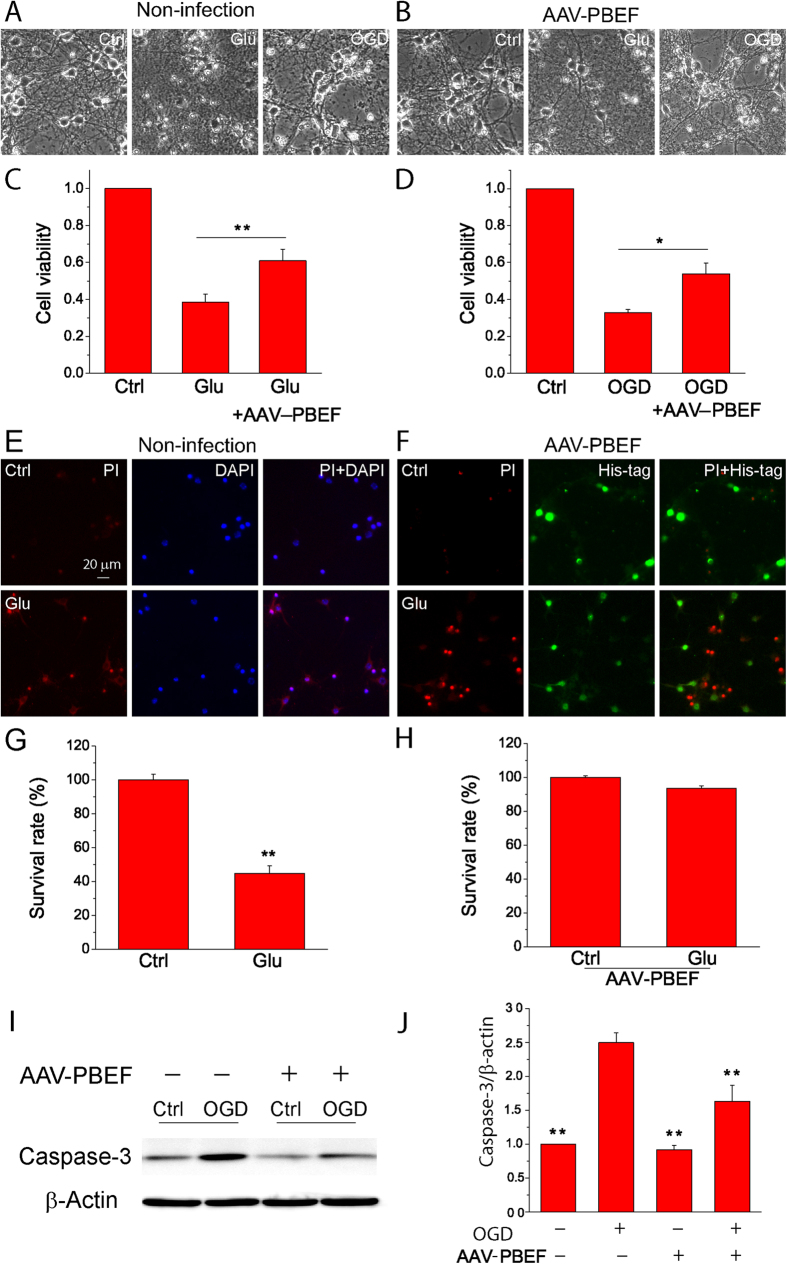
Overexpression of PBEF by AAV infection reduces neuronal death and caspase-3 activation after glutamate and OGD stimulations. (**A,B**) Phase contrast images of cortical neurons without (**A**) and with (**B**) AAV infection under conditions of control, 24 h glutamate treatment (30 μM glutamate together with 3 μM glycine) and 1 h OGD exposure followed by 24 h reperfusion. (**C,D**) Summary of neuronal viability after glutamate treatment (**C**) and OGD (**D**) by MTT assay. Data are shown as mean ± SE; n = 3–5 independent experiments. **P < 0.01 versus Glu, *P < 0.05, versus OGD ANOVA test. (**E,F**) Fluorescent images of double staining of PI and DAPI or PI and His-tag in neurons without (**E**) and with (**F**) AAV infection under conditions of control and glutamate treatment. (**G,H**) Summary of cell survival of neurons without (**G**) and with (**H**) AAV infection after glutamate stimulation based on PI staining. n = 3 independent experiments. **P < 0.01, t-test. **(I,J**) Western blot images of cleaved caspase-3 and β-actin (**A**) and summary data of cleaved capase-3 expression (**B**). The data were presented as the ratio of caspase-3 to β-actin and normalized to the control condition; n = 4 independent experiments. **P < 0.01 versus OGD without AAV infection (AAV-PBEF), ANOVA test.

Next, we determined whether PBEF overexpression by AAV infection could protect neurons against ischemic insults. Neuronal cultures with and without viral infection were subject to glutamate excitotoxicity and OGD stimulation. Morphologically, neurons without viral infection appeared with rounded soma and broken dendrites 24 h after glutamate or OGD stimulation, whereas for the neuronal cultures infected with the viral vectors, many neurons maintained healthy morphology (Fig. 3A,B). These protective effects were further assessed by MTT assay for cell viability. Glutamate reduced neuronal viability to 38.7 ± 4.3% of the control levels, whereas the neuronal viability was restored to 61.0 ± 6.1% of the control levels by viral infections (Fig. 3C). Similarly, neurons infected with the viral vectors exhibited a significant increase in cell viability after OGD stimulation (Fig. 3D). We also did PI staining to evaluate neuronal death after glutamate stimulation. Our results show that glutamate caused a significant increase of PI+ neurons in non-infected neuronal cultures; however, the majority of His-tag+ neurons were PI- after glutamate stimulation (Fig. 3E,F). Quantitative analyses revealed that PBEF overexpression significantly increased neuronal survival after glutamate stimulation (Fig. 3G,H). These results demonstrate that PBEF overexpression by viral infection can protect neurons against glutamate excitotoxicity and OGD.

Caspase-3 is a pivotal effector of apoptotic cell death. It exists as a precursor under normal conditions and is cleaved to activate in response to apoptotic stimulation. We next investigated the effect of PBEF overexpression on cleaved caspase-3. Western blotting analyses show that PBEF overexpression by viral infection had no effect on cleaved caspase-3 levels under normal conditions, but significantly attenuated the OGD-stimulated increase in cleaved caspase-3 levels (Fig. 3I,J). Collectively, these results demonstrate that caspase-dependent apoptotic signaling pathway also contributes to the neuroprotective effect of PBEF in ischemia.

### PBEF prevents glutamate-induced mitochondrial fragmentation

**Figure 4:**
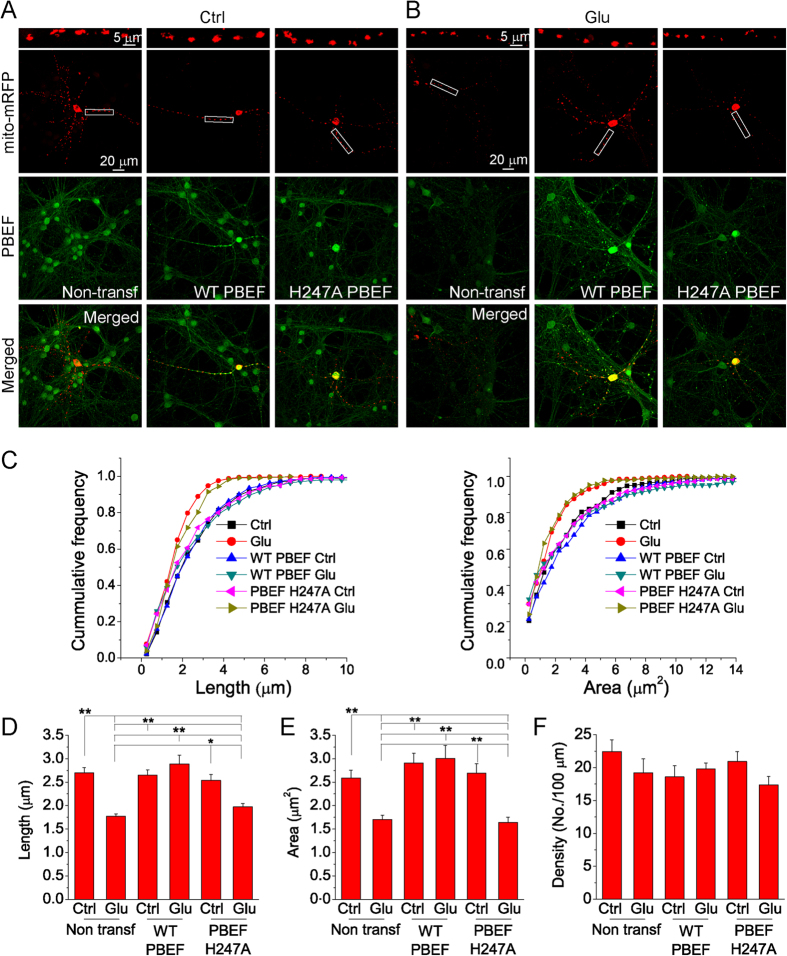
PBEF prevents mitochondrial fragmentation after glutamate stimulation. (**A**,**B**) Maximal projection confocal images of primary cortical neuron transfected with mito-mRFP alone and cotransfected with mito-mRFP and WT or H247A PBEF. Neurons were treated with or without 30 μM glutamate together with 3 μM glycine for 6 h. The upper panels are the high-resolution images of the boxed regions in the lower panels. (**C**) Cumulative frequency distribution curves of mitochondrial length and area under different conditions. The values of histogram intervals (bins) are 0.25 μm for mitochondrial length and 0.5 μm^2^ for mitochondrial area. The data show that glutamate treatment caused significant increase in the number of short and small mitochondria and this effect was inhibited by the overexpression of WT PBEF, but not by H247A PBEF. (**D–F**) The summary of average mitochondrial length, area and density in the dendrites for different conditions. *P < 0.05, **P < 0.01, ANOVA test. Data were collected from 10–15 neurons and a total of 220–326 mitochondria for each condition. Results are shown as mean ± SE; **P < 0.01, *P < 0.05, versus Non-transf with Glu and H247A with Glu, ANOVA test.

Mitochondria play a major role in both necrotic and apoptotic cell death in acute and chronic neurodegenerative diseases^22^,^23^,^24^. Mitochondrial fragmentation has been widely detected during excitotoxicity-induced apoptosis and plays an essential role in the mediation of mitochondrial dysfunction. Glutamate stimulation leads to shortening of mitochondria in a time dependent manner^15^,^25^. Thus, next we tested whether PBEF could prevent or suppress glutamate-induced mitochondrial fragmentation and whether this effect depended on its enzymatic activity of NAD^+^ synthesis. To visualize mitochondria, we transfected neurons with pCAGGS-mito-mRFP alone or co-transfected neurons with pCAGGS-mito-mRFP and pCAGGS-WT PBEF or with pCAGGS-mito-mRFP and pCAGGS-H247A PBEF. Two days after transfection, neurons were treated with 30 μM glutamate for 6 h and fixed for evaluation of mitochondrial length, area and density^15^. Confocal images of transfected neurons revealed individual mitochondria in neuronal dendrites under control conditions, but mitochondrial fragmentation occurred after glutamate exposure (Fig. 4A). However, mitochondria in neurons co-transfected with pCAGGS-mito-mRFP and pCAGGS-WT PBEF exhibited comparable length and area after glutamate stimulation with mitochondria in neurons without glutamate stimulation (Fig. 4B). Frequency distribution analysis revealed that glutamate induced an increase in the number of fragmented mitochondria with short length and small area, but the overexpression of WT PBEF could prevent this effect, while overexpression of H247A PBEF had no effect on these parameters (Fig. 4C, Supl Fig. 4). Statistically, WT PBEF, but not H247A PBEF, significantly inhibited glutamate-induced reductions of mitochondrial mean length and area in neurons (Fig. 4D,E). The density of mitochondria in dendrites, however, was affected by neither WT nor the mutant PBEF (Fig. 4F). These results demonstrate that PBEF prevents glutamate-stimulated mitochondrial fragmentation through its enzymatic activity. Consistently, the replenishment of NAM reduced glutamate-induced mitochondrial fragmentation (Supl Fig. 5).

### Overexpression of PBEF suppresses the impairment of mitochondrial biogenesis during glutamate and OGD stimulations

**Figure 5:**
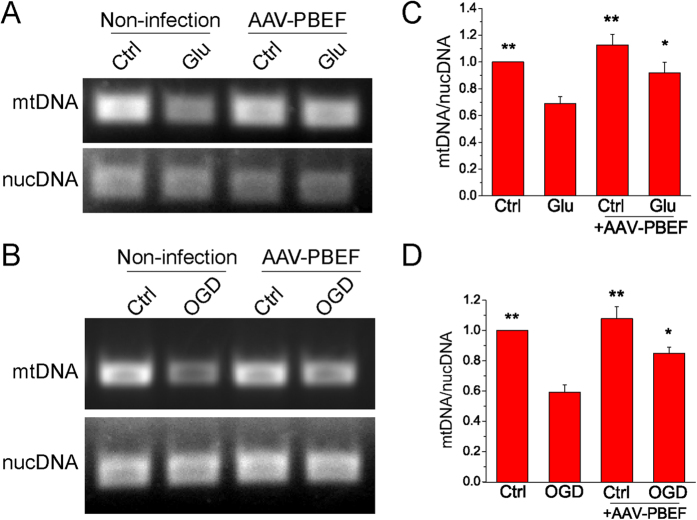
Overexpression of PBEF by viral transduction reduces mtDNA loss after glutamate and OGD stimulations. (**A,B**) Agarose gel images of mtDNA and nucDNA. Neurons without and with AAV infection were treated with 3 μM glutamate together with 0.3 μM glycine for 24 h (**A**) or were exposed to 1 h OGD followed by 24 h reperfusion (**B**). (**C,D**) Quantification of mtDNA amount after glutamate (**C**) and OGD (**D**) stimulations. The data were presented as the ratio of mtDNA/nucDNA and normalized to the control condition using nucDNA as an internal control. Notice that the overexpression of PBEF by viral infection prevented the glutamate-induced decrease of mtDNA content. Data are shown as mean ± SE; n = 3 independent experiments. (**C**) **P < 0.01 versus Glu, ANOVA test. (**D**) **P < 0.01, *P < 0.05 versus OGD, ANOVA test.

**Figure 6:**
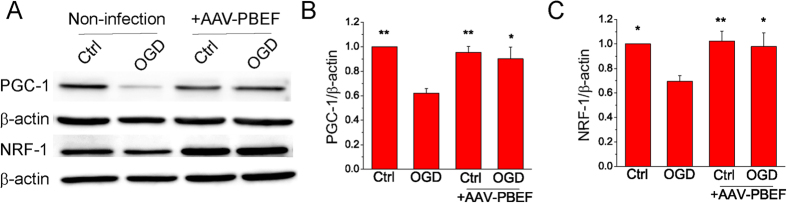
Overexpression of PBEF by viral transduction suppresses OGD-induced impairment of neuronal mitochondrial biogenesis. (**A**) Western blot images for PGC-1, NRF-1 and β-actin in control and OGD stimulated primary neuronal cultures. The neuronal cultures without and with AAV infection were subjected to OGD for 1 h followed by 24 hreperfusion. (**B,C**) Summary of quantitative analysis of PGC-1 (**B**) and NRF-1 (**C**) expression levels. The data were presented as the ratio of the proteins to β-actin and normalized to the control condition. n = 4 independent experiments. **P < 0.01, *P < 0.05 versus OGD, ANOVA test.

Improved mitochondrial biogenesis has been reported to reduce ischemic injury in *in vitro* and *in vivo* models^26^. Our recent studies indicate that NAD^+^ can significantly attenuate the impairment of mitochondrial biogenesis after glutamate and OGD stimulations^7^,^15^. To further investigate the mechanism by which PBEF suppresses the impairment of mitochondrial biogenesis, we first determined the effect of PBEF on the mitochondrial DNA (mtDNA) content, which represents mitochondrial mass, after glutamate and OGD stimulations. Primary neuronal cultures with or without viral infection were stimulated with glutamate for 24 h or subjected to 1 h OGD, and collected 24 h later for quantification of mtDNA using qPCR. Nuclear DNA (nucDNA) was used as an internal control. We observed a significant decrease of mtDNA/nucDNA ratio after glutamate excitotoxicity and OGD in cultured neurons without PBEF overexpression, but the decrease of mtDNA content was inhibited when neurons were overexpressed with WT PBEF by viral infection (Fig. 5A–D). Next, we examined the effect of PBEF on PGC-1, a master regulator of mitochondrial biogenesis, and its downstream effector NFR-1, after OGD. Consistently, the protein levels of PGC-1 decreased significantly by OGD stimulation, but PBEF overexpression by viral transduction markedly suppressed this OGD-induced reduction (Fig. 6A,B). Similarly, OGD also caused a significant decrease of NRF-1 levels, which was attenuated by PBEF overexpression (Fig. 6A,C). Taken together, these results demonstrate that the overexpression of PBEF can attenuate ischemia-induced mitochondrial damage and impairment of mitochondrial biogenesis.

## Discussion

In the current study, using *in vitro* ischemic models of primary cultured mouse cortical neurons, molecular biology, neuronal death assay, fluorescent imaging, mitochondrial biogenesis measurements, we have provided several lines of evidence demonstrating that PBEF, through its enzymatic activity of NAD^+^ synthesis, can ameliorate neuronal death and mitochondrial damage after ischemia. First, PBEF can ameliorate apoptotic neuronal death after glutamate and OGD stimulations. Second, PBEF can inhibit AIF translocation and caspase-3 activation after glutamate excitotoxicity. Third, PBEF can inhibit glutamate-induced mitochondrial fragmentation. Fourth, PBEF can reduce glutamate and OGD-induced loss of mitochondrial mtDNA content. Fifth, PBEF can suppress mitochondrial fragmentation and the impairment of mitochondrial biogenesis. The mutant PBEF, with little NAD^+^ synthetic activity, did not display similar beneficial effects in ischemia. Our study has demonstrated not only that the enzymatic activity of NAD^+^ biosynthesis is required for the neuronal protective effect by PBEF, but also indicates that the enzymatic activity is critical to improve mitochondrial biogenesis and function in ischemia.

Ischemia triggers apoptosis intrinsically by both caspase-3 independent pathway involving AIF translocation and caspase-3 dependent pathway with mitochondrial cytochrome C release^27^,^28^. On the other hand, ischemia causes the reduction of NAD^+^ levels in mouse and cultured neuron models^6^,^7^,^12^. NAD^+^ depletion leads to translocation of AIF from mitochondria to nucleus^19^. The release of AIF from mitochondria induces peripheral chromatin condensation, large-scale DNA fragmentation and eventually results in caspase-independent apoptotic cell death^20^. AIF translocation from mitochondria to nuclei is a prominent phenomenon of apoptosis and inhibition of AIF translocation promotes neuronal survival in hippocampal CA1 region after transient forebrain ischemia^29^. Therefore, prevention of AIF translocation can protect neurons from cell death. Previous studies including from our laboratory have shown that exogenous replenishment of NAD^+^ and NAM are neuroprotective and can prevent AIF translocation during *in vitro* and *in vivo* ischemic insults^7^,^13^,^14^,^15^. As the rate-limiting enzyme in the mammalian NAD^+^ biosynthesis salvage pathway, PBEF is critical in modulating cellular NAD^+^ homeostasis and energy metabolism. Our results from this study showing that overexpression of WT PBEF, but not enzymatic activity deficient mutant PBEF, can inhibit AIF translocation further consolidate the role of NAD^+^ in energy metabolism and the inhibition of AIF translocation after ischemia.

Poly (ADP-ribose) polymerase-1(PARP-1), an NAD^+^ consuming enzyme, is overactivated and plays a dominant role in neuronal death after ischemic stroke^30^,^31^. The over-activation of PARP-1 leads to a significant NAD^+^ depletion and induces downstream release of AIF from mitochondria to the nucleus, which results in a caspase-independent pathway of apoptotic cell death^32^. Studies also showed that N-methyl-N’-nitro-N’-nitrosoguanidine (MNNG), an alkylating agent, induced PARP-1 activation triggers AIF translocation in neurons^32^,^33^,^34^. Cells expressing reduced levels of PBEF or exposed to a potent PBEF inhibitor, FK866, displayed an increased sensitivity to cell death induced by the over-activation of PARP-1 in response to the MNNG stimulation in T and B lymphocytes^35^. However, NAD^+^ also increases PARP-1 activity, suggesting that NAD^+^ depletion lies upstream of mitochondrial AIF translocation. Our data indicate that the promotion of neuronal survival and prevention of AIF translocation by PBEF after glutamate excitotoxicity are resulted from preserving high intracellular NAD^+^ rather than the activation of PARP-1 *per se*. In the present study, we also showed that AAV-mediated PBEF overexpression significantly reduced caspase-3 activation during OGD stimulation in primary neuronal cultures. This finding is consistent with results showing that inhibition of PBEF by its inhibitor FK866 increases the expression of cleaved caspase-3 in OGD model of cultured neurons and recombinant PBEF administration through intracerebroventricular injection decreases caspase-3 activity in the rat hippocampal CA3 region after transient global cerebral ischemia^8^,^36^. Thus our study has established that PBEF can protect against apoptotic neuronal death through both caspase-independent and caspase-dependent pathways.

Mitochondrial length, size and shape are controlled by fission and fusion^23^,^37^. Under physiological conditions, neurons generate new mitochondria through the coordinated action of transcriptional factors and co-activators to maintain the balance between fission and fusion. The balance is disrupted in neuronal injury and degeneration, thus causing morphological change and structural damage associated with mitochondrial dysfunction^38^,^39^,^40^. Mitochondrial fragmentation is an early event that occurs before the release of mitochondria-dependent apoptotic proteins and is the key mechanism in glutamate excitotoxicity and AIF translocation mediated cell death^41^. In the present study, we found that overexpression of WT PBEF, not mutant PBEF, prevented glutamate-induced reductions of mitochondrial length and area, but interestingly did not affect the density of mitochondria. This protective effect is consistent with our recent observation that extracellular replenishment of NAD^+^ suppresses mitochondrial fragmentation and AIF translocation during glutamate challenge^15^. Fragmented mitochondria can be engulfed and degraded by autophagosomes^42^. It was reported that the overexpression of PBEF significantly promotes neuronal survival through increasing autophagy in both *in vivo* and *in vitro*^9^; therefore, it is plausible that PBEF could facilitate the clearance of fragmented mitochondria via regulation of mitophagy during ischemia.

In the present study, we also found that overexpression of PBEF attenuated the decrease of the relative amount of mtDNA to nucDNA after glutamate and OGD stimulations. These results are in agreement with previous studies showing that NAD^+^ is highly correlated with mitochondrial biogenesis^15^,^16^,^17^,^18^,^43^ and that inhibition of mtDNA loss restored mitochondrial biogenesis and reduced infarct size in a mouse permanent MCAo model^26^. PGC-1 and NRF-1 are the key regulators of mitochondrial biogenesis^16^,^26^,^44^, and up-regulations of them protect neurons against OGD-induced injury^44^. Our data showed that PGC-1 and NRF-1 levels were decreased after OGD but the overexpression of PBEF restored their levels. Our results that PBEF could prevent the loss of mitochondrial mass and the impairment of mitochondrial biogenesis, but did not affect mitochondrial number, suggest that PBEF may not break the balance between mitochondrial fusion and fission. These findings are in line with our previous observations that exogenous NAD^+^ suppressed glutamate-induced reduction in mtDNA content and PGC-1 and NRF-1 levels, and mitochondrial length and area, but not mitochondrial density in the dendrites^7^,^15^. In the future study, it will be interesting to explore whether suppression of the impairment of mitochondrial biogenesis by PBEF overexpression is a consequence of the changes in the expression of mitochondrial fission and fusion proteins such as dynamin-related protein-1 (DRP-1) and mitofusin-1 (mfn-1)^45^,^46^,^47^,^48^.

At present, there is no evidence to support any direct interactions between PBEF and PGC-1 or NRF-1, but considerable evidence suggests that PBEF-mediated NAD^+^ biosynthesis increases Sirt1 activity in diverse subcellular compartments^8^,^49^. As an NAD^+^ dependent histone deacetylase, Sirt1 plays an important role in the regulation of mitochondrial biogenesis through directly activating PGC-1 by deacetylation and indirectly promoting downstream NRF-1 nuclear translocation via the modulation of AMPK and Akt signaling pathways^50^,^51^. Since Sirt1 is an NAD^+^ -consuming enzyme and PBEF is a rate-limiting enzyme for NAD^+^ synthesis, PBEF-mediated improvement in mitochondrial biogenesis after ischemic stimulation is likely through Sirt1 signaling pathway.

The observed beneficial effects of PBEF in the present study might also be related to other mitochondrial signaling pathways that trigger neuronal death during ischemic injury. For example, in the myocardial injury model, PBEF can delay or inhibit mitochondrial permeability transition pore (mPTP) opening both *in situ* murine heart and the isolated murine cardiomyocytes^52^. Meanwhile, PBEF was found to inhibit apoptosis and prevent metabolic dysfunction via regulating the expression of mitochondrial-dependent anti-apoptotic protein Bcl-2, as well as pro-apoptotic proteins, such as Bax and cytochrome C^36^. These regulations depend on the activation of pro-survival kinases, PI3K/AKT and MEK1/2, mediated-signaling pathways. Although our previous study showed that PBEF can reduce MMP depolarization^7^, further investigations are needed to clarify if these possible molecular mechanisms are involved in the PBEF-mediated neuronal protection during ischemia.

In summary, the important findings of the current study are that PBEF can ameliorate apoptotic neuronal death through caspase-dependent and independent pathways, and PBEF can suppress the loss of mitochondrial mass and impairment of mitochondrial biogenesis in ischemia by inhibiting mitochondrial fragmentation and rescuing the key molecules involving mitochondrial biogenesis. These beneficial effects of PBEF require the enzymatic activity of NAD^+^ synthesis. Our study revealed a previously unknown mechanism for the neuroprotective effect imposed by PBEF in ischemia. Together with previous studies from ours and other laboratories, it indicates that PBEF can exert a neuronal and brain protective effect in ischemia through different mechanisms. Our results provide novel insights into the mechanisms of neuronal and brain protection in ischemia and indicate that enhancing cellular energy metabolism through NAD^+^ salvage pathway and inhibiting mitochondrial damage are valuable neuroprotective strategies for ischemic stroke.

## Methods

### Animals

Embryos from pregnant C57BL/6 J female mice were used for the preparation of primary cortical cultured neurons. All procedures were performed according to the NIH Guide for the Care and Use of Laboratory Animals and were approved by the University of Missouri Animal Care Quality Assurance Committee.

### Primary neuronal cultures and *in vitro* ischemia models

Primary mouse cortical neuronal cultures were prepared from embryonic day 15/16 (E15/16) C57BL/6J mice as previously described^7^,^15^. Briefly, cortical tissues of embryos were dissociated by mild mechanical triturating after digestion with trypsin. The isolated cells were plated onto poly-L-lysine-coated (50 or 100 μg/mL) (Sigma-Aldrich, MO, USA) tissue culture plates or glass coverslips of 12 mm in diameter (Fisher Scientific, IL, USA) in a culture plate with Dulbecco’s modified Eagle medium (DMEM)/F12 (Life Technologies, CA, USA) supplemented with 10% heated-inactivated fetal bovine serum (FBS) (Atlanta Biological, GA, USA) for 5 h, and the medium was then changed to Neurobasal Media (NBM) (Invitrogen) containing 2% B-27 serum free supplements (Life Technologies), 0.5 mM L-glutamine (Life Technologies) and 50 U/ml penicillin/streptomycin (Life Technologies). In order to suppress the growth of glial cell and increase the purity of neuronal cultures, 5 μM cytosine β-D-arabino-furanoside (Sigma-Aldrich) was added into medium 24 h after plating. Our procedure yielded high purity of cultured neurons. The cultures were maintained with a 50% medium change every 3 days at 37 °C in an incubator with a humidified atmosphere of 5% CO_2_ and 95% air. Experiments were conducted between 10–14 days *in vitro* (DIV) as the neurons were matured at this age and vulnerable to glutamate-induced excitotoxicity.

To mimic ischemic conditions *in vitro*, primary neurons were exposed to OGD or stimulated with excitotoxic glutamate. Transient OGD was induced similar to previous reports^7^,^53^. The medium in cultured neurons in 24-well plates was removed, rinsed out for three times and replaced with serum- and glucose-free balanced salt solution (BSS), pH 7.4, containing (mM)^54^: NaCl 116, CaCl_2_ 1.8, MgSO_4_ 0.8, KCl 5.4, NaH_2_PO4 1, NaHCO_3_ 14.7, HEPES 10. The culture plates were then placed within an anaerobic chamber in a 37 °C incubator. The chamber was sealed and flushed with 99% N_2_ and 1% air for 60 min controlled by a HypoxyDial (STARR Life Sciences, PA, USA). After OGD, the BSS was replaced by normal feeding medium and neurons were then returned to 5% CO_2_ and 95% air for 24 h. Control neurons were replaced with BSS containing 5.5 mM glucose and incubated under normoxic condition. To induce glutamate excitotoxicity, cultured neurons were exposed to glutamate and glycine by directly adding them into culture wells for different times as described in different experiments^7^,^15^.

### Transfection of DNA in primary cultured neurons

Neurons were transfected at 6 DIV using lipofectamine 2000 reagent (Life Technologies) as in previous studies^7^,^15^. Before transfection, neurons were replenished with regular growth medium without antibiotics. For transfection of neurons in each well in 24-well plates, 0.8 μg DNA plasmid was diluted in 50 μL serum-free NBM and gently mixed with 50 μL NBM containing 2 μL lipofectamine 2000 reagent (Life Technologies). After incubation for 20 min at room temperature, DNA and lipofectamine 2000 complexes were added drop by drop to the cultured neurons in the well. After incubation for 6 h in the incubator, the medium was replaced by normal NBM growth medium containing penicillin/streptomycin. The neuronal cultures were ready for experiments 48 h later. Similar to our previous studies^7^,^55^, we used DNA plasmids with a CAGGS promoter (pCAGGS) encoding EGFP, wild type (WT) and mutant hPBEF (i.e., H247A and H247E), mRFP with a mitochondria-targeting sequence (mito-mRFP) cDNAs (see Figs 1, 2 and 4).

### Infection of primary cultured neurons by adeno-associated virus (AAV) vectors

Compared with other types of viral vectors, AAV vectors showed higher packaging and delivering efficiency with minimal cytotoxicity or inflammation for expressing transgenes in neuronal cultures. Primary neuronal cultures were infected with serotype 2/9 AAV vectors encoding human WT PBEF with a His-tag at C-terminal under the control by the CMV promoter, *i.e.*, AAV2/9-CMV-PBEF vectors. The vectors were added to neuronal cultures at 3–5 DIV in serum-free NBM medium with a genome copy (GC)/cell ratio of 3 × 10^5^ for 6 h. The medium was then replaced by normal NBM growth medium. The neurons were cultured for at least one week before the experiments. The overexpression of PBEF in neurons was confirmed by immunocytochemistry and Western blot analysis.

### Immunostaining and analyses

The procedures were described as in our previous studies^7^,^15^. Neurons cultured on the poly-L-lysine coated-glass coverslips were washed with PBS (0.01 M) three times, fixed with ice-cold 4% paraformaldehyde (PFA) in PBS for 15 min, and then rinsed carefully for three times with PBS. Neurons were then permeabilized with ice-cold 0.1% Triton X-100 in PBS for 10 min and blocked with 5% serum in PBS for 1 h at room temperature. Neurons were then incubated with primary antibodies rabbit anti-PBEF polyclonal antibody (Bethyl Laboratories, TX, USA), rabbit anti-AIF polyclonal antibody (Sigma-Aldrich), mouse anti-His tag monoclonal antibody (Millipore, CA, USA) in PBS containing 1% serum, 0.1% Triton X-100 at 4 °C overnight. The neurons were washed with PBS for 5 min three times and then incubated with fluorescently labeled secondary antibodies rhodamine-conjugated donkey anti-rabbit IgG (Millipore), FITC-conjugated goat anti-rabbit IgG (Millipore), Alexa fluor-488-conjugated donkey anti-mouse IgG (Life Technologies) in 1% serum, 0.1% Triton X-100 in PBS for 1 h in the dark at room temperature. The nuclei were counterstained with DAPI. The samples were subjected to fluorescence detection using a Nikon FN1 epi-fluorescence microscopy equipped with a CoolSNAP-EZ CCD camera (Photometrics, AZ, USA) or an Olympus confocal microscope, and analyzed by MetaMorph software (Molecular Devices, CA, USA). For all the three independent experiments, 6–8 fields per glass coverslips and duplicate or triplicate glass coverslips for each independent experiment were evaluated.

### Neuronal death assay

We used terminal dinucleotidyltransferase-mediated UTP end labeling (TUNEL) staining to identify apoptotic neuronal death after glutamate stimulation^15^,^56^. Cultured neurons were fixed with a freshly prepared 4% PFA in PBS for 30 min, incubated with permeabilisation solution (0.1% sodium citrate in 0.1% Triton X-100) for 2 min on ice, and subsequently with TUNEL reaction mixture (*In Situ* Cell Death Detection Kit, Fluorescein; Roche, IN, USA) in a humidified environment for 1 h at 37 °C in the dark. Samples were directly analyzed under a Nikon FN1 epi-fluorescence microscopy using a 40× magnification objective. The total number of neurons was counted based on DAPI stained nuclei and the number of apoptotic neurons was counted based on TUNEL+ cells. The counting for all the three independent experiments was performed by an experimenter blind to the experimental conditions. The neuronal survival rate was defined as the ratio of the number of TUNEL- cells to the number of DAPI-stained nuclei.

Propidium iodide (PI) (Sigma-Aldrich) staining was also performed to evaluate neuronal death^7^,^15^. Neurons cultured on the glass coverslips were washed two times with PBS, and incubated with PI (5 μg/mL) for 30 min at 37 °C. Neurons then were washed with PBS for three times and fixed with 4% PFA for 20 min at room temperature. Nuclei were counterstained with DAPI.

Neuronal cell viability was assessed by MTT assay (Thiazolyl Blue Terazolium Bromide) (Sigma-Aldrich)^7^,^15^. MTT (5 mg/ml in PBS and filter sterilized) was added to neurons cultured in 48-well plates and incubated at 37 °C for 4 h in a 5% CO_2_ atmosphere. The MTT-containing medium was replaced with dimethyl sulfoxide (Sigma-Aldrich) to dissolve the formed blue formazen. The absorbance was read at 540 nm with a 96-well microplate reader (Molecular Device, CA, USA).

### Measurement of mitochondrial morphology

In order to study the effect of PBEF on glutamate-induced mitochondrial fragmentation, neurons expressing with mitochondria-targeted mRFP (mito-mRFP) were imaged using an Olympus confocal microscope with a 60× oil immersion objective. Mitochondrial length and area were analyzed from the complete series of z-stack confocal images using MetaMorph offline software^15^. All the images were adjusted to the same scale and user-defined thresholds for pixel intensity and object size were used to measure mitochondrial area and length. For each neuron, mitochondria located within 100 μm in dendrite from soma were selected for analysis.

### Mitochondrial DNA (mtDNA) quantification

qPCR was used to amplify mtDNA and nucDNA as described before^7^,^15^. Briefly, the total DNA of cultured neurons was extracted and purified using the genomic DNA extraction kit (Qiagen Sciences, CA, USA). The total DNA derived from neurons in one well from 12-well plates was added to the polymerase chain reaction (PCR) mixture with GoTaq Flexi DNA Polymerase (Promega, WI, USA). The PCR products were separated by a 1% agarose gel and stained with ethidium bromide (0.5 mg/mL). The images were acquired by scanning with a Fuji LAS 3000 densitometer (Fuji, Tokyo, Japan). The densities of the mtDNA and nucDNA bands were calculated using ImageJ software. The density ratio of mtDNA to nucDNA was used to determine the relative mtDNA content and mitochondrial mass.

### Western blot analysis

Western blot was used to analyze PBEF, His-tag, (peroxisome proliferator-activated receptor gamma coactivator 1 (PGC-1) and nuclear respiratory factor-1 (NRF-1) expression levels in neuronal cultures. Protein samples were separated by 10% SDS-polyacrylamide gel electrophoresis and transferred to PVDF membranes. The membranes were blocked by 5% non-fat dry milk and incubated with rabbit anti-PBEF polyclonal antibody (1:1000, Bethyl Laboratories), mouse anti-His-tag monoclonal antibody (1:1000, Millipore), rabbit anti-PGC-1 polyclonal antibody (1:500, Santa Cruz Biotechnology, CA, USA), rabbit anti-NRF-1 polyclonal antibody (1:500, Santa Cruz Biotechnology) and HRP-conjugated second antibody. The protein bands were visualized by ECL Western blotting detection system. β-actin was used as a control for equal loading of the total protein.

### Statistical analysis

All data were expressed as means ± SEM obtained from three or more independent experiments using cortical neuronal cultures isolated from different mice. Statistical comparisons were made by t-test for two groups and a one-way ANOVA test followed by Tukey test for multiple groups. P-value < 0.05 or < 0.01 was considered to be statistically significant and indicated as one or two stars respectively.

## Additional Information

**How to cite this article**: Wang, X. *et al*. Pre-B-cell colony-enhancing factor protects against apoptotic neuronal death and mitochondrial damage in ischemia. *Sci. Rep.* **6**, 32416; doi: 10.1038/srep32416 (2016).

## Supplementary Material

Supplementary Information
