# Supplementary material for: Pre-B-cell colony-enhancing factor protects against apoptotic neuronal death and mitochondrial damage in ischemia
Source: Sci Rep. 2016 Aug 31;6:32416. doi: 10.1038/srep32416 (PMC5006239; doi:10.1038/srep32416)
Supplement: Supplementary Information [file srep32416-s1.pdf]

Pre-B-cell colony-enhancing factor protects against apoptotic neuronal death and mitochondrial damage in ischemia

Xiaowan Wang[1], Hailong Li[1,2], Shinghua Ding[1,2]

[1]Dept. of Bioengineering, [2]Dalton Cardiovascular Research Center

University of Missouri, Columbia, MO 65211

Supplementary Information

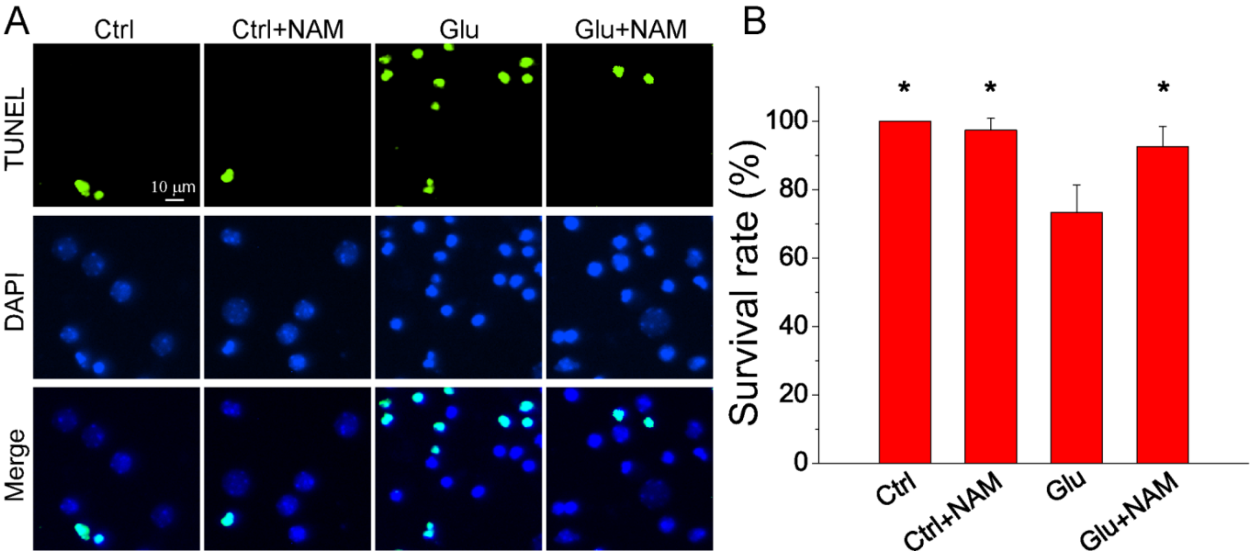

Supplemental Figure 1. Exogenous NAM ameliorates neuronal apoptosis after glutamate stimulation. (A) Fluorescent images of TUNEL and DAPI staining in neurons. Neuronal cultures were treated with 30 μM glutamate and 3 μM glycine for 24 h in the presence and absence of 15 mM NAM. (B) Neuronal survival rates under different conditions. Data were obtained from n=3 independent experiments. **P*<0.05 versus Glu, ANOVA test.

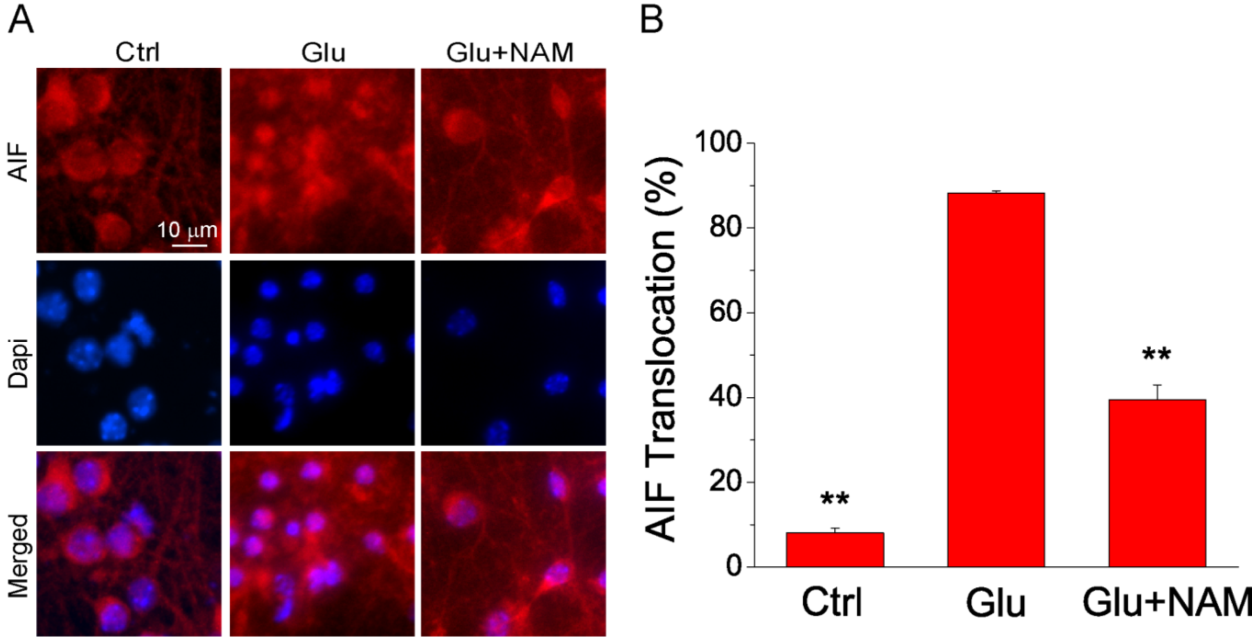

Supplemental Figure 2. NAM prevents the translocation of AIF from mitochondria to the nucleus after glutamate excitotoxicity. (A) Fluorescent images of neurons stained with AIF antibody and DAPI. Neurons were treated with 100 μM glutamate together with 10 μM in the absence and presence of 15 mM NAM for 3 h. Translocation of AIF from mitochondria to nuclei is illustrated by the overlap of AIF (red) and DAPI (blue) signals. (B) Summary of AIF translocation after glutamate stimulation. Data was quantified by the number of cells with overlap of AIF and DAPI out of the total number of cells determined by DAPI staining. Data were obtained from n=3 independent experiments. ***P*<0.01 versus Glu, ANOVA test.

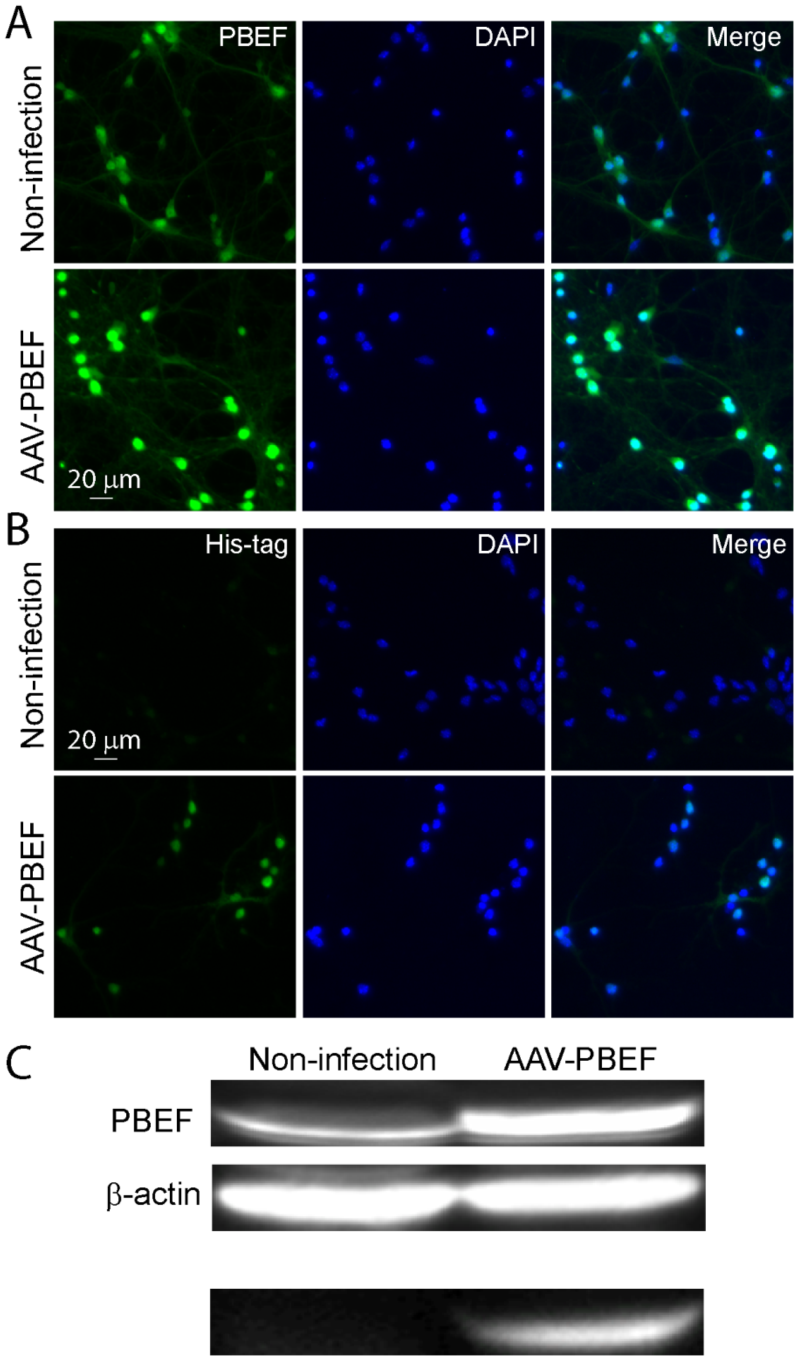

Supplementary Figure 3. Overexpression of PBEF in neurons by AAV infection. A-B) Fluorescent images of infected and non-infected primary neurons stained with His-tag (A) and PBEF (B) antibodies. C) Western blot images of PBEF and β-actin from infected and non-infected neuronal cultures.

Supl Fig 4

A

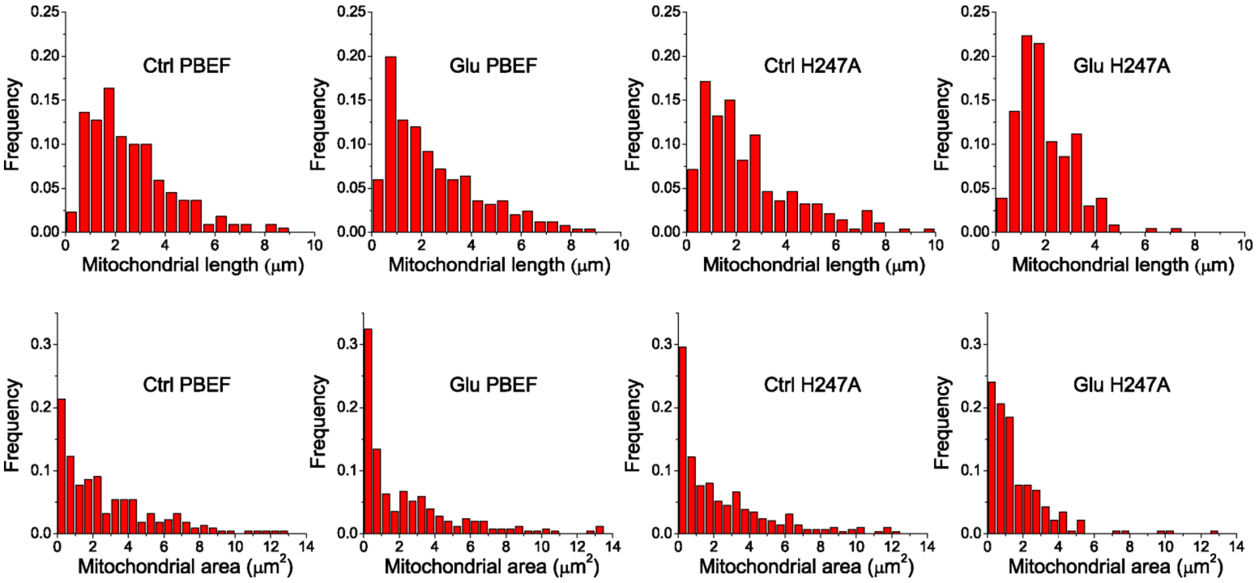

Supplemental Figure 4. The effect of PBEF on the frequency distribution of mitochondrial length and area after glutamate treatment. Frequency distributions of neuronal mitochondrial length (A) and area (B) with and without treatment of neurons with 30 μM glutamate and 3 μM glycine for 6 h. The values of histogram interval (bin) are 0.25 μm for mitochondrial length and 0.5 μm^2 for mitochondrial area. Notice that PBEF effectively prevented the decrease of mitochondrial length and area induced by glutamate stimulation.

Supl Fig 4

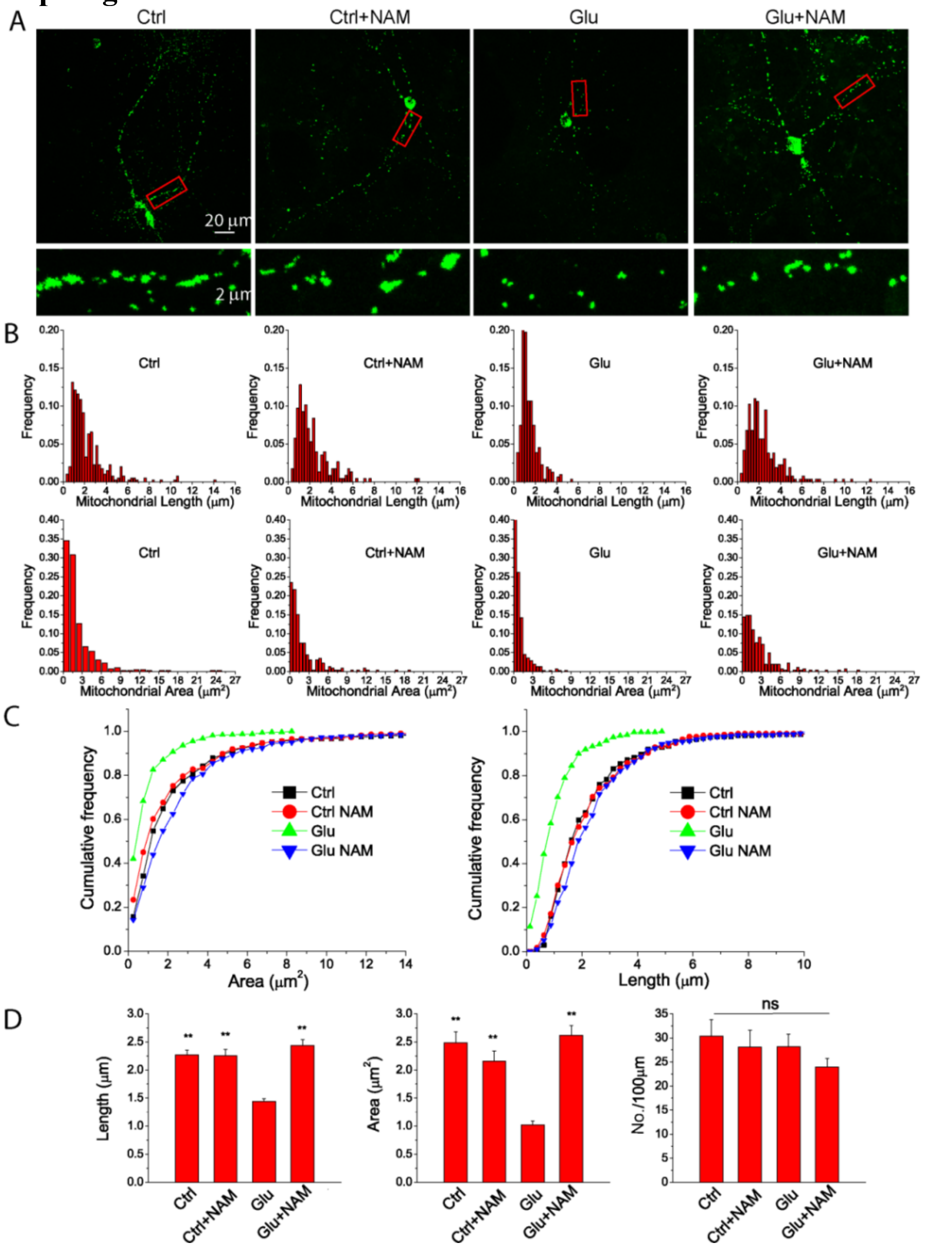

Supplemental Figure 5. Exogenous NAM inhibits glutamate-induced mitochondrial fragmentation. (A) Maximal projection confocal images of neurons transfected with mito-AcGFP1. Neurons were treated without or with 30 µM glutamate together with 3 µM glycine for 24 h in the absence and presence of 15 mM NAM. The lower panels are the high-resolution images of the boxed region in the upper panels. (B) Cumulative distribution curves of mitochondrial length and area under different conditions. The values of histogram interval (bin) are 0.25 µm for mitochondrial length and 0.5 μm^2 for mitochondrial area. Notice that glutamate treatment caused a significant increase in the number of small mitochondria and this effect was attenuated by NAM supplementation. (C-E) The summary of average mitochondrial length, area and density for each condition. Notice the density of mitochondria in neuronal dendrites was not affected by exogenous NAM. Data were collected from 10-15 neurons and a total of 226-396 mitochondria in each condition. Results are shown as mean ± SE; ***P*<0.01 versus Glu, ANOVA test.
